# The Efficacy of Two Group Interventions on Mental Representations, Attachment Security, and Trauma Symptoms in Ethnically and Socioeconomically Minoritized Young Adolescents in an Urban Middle School

**DOI:** 10.3390/ijerph20105789

**Published:** 2023-05-11

**Authors:** Geoff Goodman, Bryan Blum, Carla Rentrop, Norka Malberg, Palakrajiv Agrawal

**Affiliations:** 1 Emory School of Medicine, Emory University, Atlanta, GA 30322, USA; 2 Clinical Psychology Doctoral Program, Long Island University, Brooklyn, NY 11201, USA; 3 Institute for Psychoanalytic Training and Research, New York, NY 10128, USA; 4 Yale Child Study Center, New Haven, CT 06519, USA; 5 Realization Center Inc., Brooklyn, NY 11201, USA

**Keywords:** young adolescents, group intervention, mental representations, attachment security, childhood trauma symptoms

## Abstract

Symptoms resulting from childhood trauma can negatively impact socioemotional well-being and school performance during early adolescence unless positive changes in attachment security and mental representations of significant relationships occur. A sample of 109 eighth grade urban students were randomly assigned to one of two weekly, one-hour, school-based group interventions—Storytelling/Story-Acting for Adolescents (STSA-A) or Mentalization-Based Treatment Group Intervention (MBT-G). The Object Relations Inventory (ORI), Adolescent Attachment Questionnaire (AAQ) and Child PSTD Stress Scale (CPSS) were administered to students and their primary group leaders at the beginning (October) and end (May) of the intervention protocol as outcome variables. Participants in both the STSA-A and MBT-G intervention conditions experienced significant increases in attachment security and decreases in trauma symptoms. Over the course of eight months of group intervention, affective valence of paternal mental representations significantly decreased for boys and for participants in the STSA-A condition, while affective valence of primary group leader mental representations significantly decreased for participants in the MBT-G condition. STSA-A and MBT-G were found to be efficacious at improving attachment security and reducing trauma symptoms in young adolescents. The strengths of each group intervention for addressing interpersonal issues unique to specific types of adolescents are discussed.

## 1. Introduction

It is estimated that 45% of US children experience at least one adverse childhood experience [1]. These experiences, known in the literature as Adverse Childhood Experiences (ACEs; [2]), can have a serious negative effect on the development of many young people during key phases of their growth. ACEs do not necessarily lead to the emergence of mental health issues, but they do indicate which individuals are more likely to end up suffering from symptoms that stem from these traumatic events [2]. In fact, the ACE scale was positively correlated with trauma symptoms among youth ages 10–17 [3] as well as depression and negative health effects [4]. Examples of common ACEs include, but are not limited to, family dysfunction, criminal activity, financial difficulty, psychological mistreatment, sexual abuse, and neglect that happened in the past [5]. PTSD, as described by [6], is “a psychiatric disorder involving development of disturbing/distressing symptoms after exposure to a traumatic event and is associated with considerable functional impairment and comorbidity” (p. 2). Individuals with PTSD might experience recurrent memories of the traumatic event, try to avoid activities or situations that might remind them of the event, and have difficulty concentrating or feeling overly alert [7]. Approximately 16% of children who experience a traumatic event before they reach adulthood will meet the criteria for a diagnosis of PTSD [8].

Although some initiatives have been taken to develop resilience programs for those exposed to potentially traumatic events, there is still no agreement in the psychological community on the most effective way of preventing PTSD in people who have experienced trauma [9]. It can also be hard to put these resilience programs into effect for those not identified as needing mental health care [10]. Even though adolescence is a period of emotional and cognitive maturation [11], many young adolescents can be exposed to certain developmental risks and display PTSD symptoms without intervention [1]. Such symptoms can include an increase in impulsivity, leading to issues with controlling emotions and behavior, issues with cognitive functioning, dissociation, physical pain, and poor relationships and self-image [12].

It has been established that young adolescents with PTSD have an increased likelihood of displaying aggressive and impulsive behavior as well as ruminating on their trauma, leading to long-term psychological effects if not adequately addressed. Traumatic events such as abuse, neglect, and parental loss are associated with higher levels of psychotic symptoms and hospitalizations in later life relative to those who did not experience such situations [13,14]. Furthermore, PTSD is linked with adolescent cannabis use disorder [15] and high rates of concurrent substance misuse [16]. In addition, female adolescents with PTSD can have changes in neural pathways that affect emotional regulation that differs from other girls [17]. Lastly, the intensity of trauma symptoms is also related to the quality of mental representations.

### 1.1. Mental Representations and Trauma

According to Mitchell and Black (2016) [18], during infancy and childhood, people attach to the earliest primary caregivers such as parents, who serve as prototypes for future relationships. These prototypes are gradually internalized as mental representations that guide future interactions with others, both positively and negatively, based on the affective valence of these relationships [19]. Object relations theory posits that the early development of a child’s psyche involves forming relationships with others, creating mental representations of those relationships and oneself. This process is seen as being important in the development of an individual’s identity and self-concept and is thought to have a lasting impact on how they interact with others and perceive themselves [20].

The way in which one anticipates the responses they will receive from others (manifested in their mental representations) is a key factor in determining the severity of a traumatic experience. Positive mental representations can protect an individual from reacting to and recovering from trauma in an unhelpful or disabling manner. An analysis of the use of the Object Relations Inventory (ORI)—a tool used for collecting mental representations—revealed that adults with more affectively positive mental representations of themselves and others had more beneficial outcomes in both clinical and everyday life than those with more affectively negative mental representations [19]. This finding is also applicable to young adolescents. Studies have demonstrated that young adolescents suffering from PTSD have more impaired representations of themselves and a less positive view of parental relationships than those who do not have PTSD [21].

Focusing on positive mental representations in interventions for young adolescents dealing with PTSD may lead to more positive outcomes. Having positive mental representations of a supportive figure can help youth process stressful situations more quickly [22], which can result in a quicker recovery from traumatic experiences.

Regarding the self, people who have more positive mental representations of the self were able to draw comfort through self-soothing or soothing from others, in contrast to those with more negative mental representations of the self, who had more difficulty self-soothing or looking to others for comfort [23]. Additionally, individuals with severe mental illness who had more distinct mental representations of their parents and the primary group leader reported improved clinical results [24]. In addition to quality of mental representations, attachment security is also related to severity of trauma symptoms.

### 1.2. Attachment Security and Trauma

Bowlby (1973) and Ainsworth (1979) [25,26] proposed that the connection between a caregiver and an infant can have a substantial impact on the child’s future social and emotional development and relationships. According to their research, two types of attachment exist—secure and insecure. People with secure attachment have the expectation that their needs will be taken care of and tend to have more self-confidence in their relationships, which can lead to increased socio-emotional skills, cognitive abilities, physical health, and mental health [27]. On the other hand, insecure attachment can be further broken down into preoccupied, dismissing, and incoherent/disorganized attachment and is characterized by an apprehension of the caregiver and anxiety that their needs will not be met [28]. Those with insecure attachment generally have lower levels of socioemotional skills, cognitive abilities, physical health, and mental health than those with secure attachment [27]. Studies have suggested that the security of attachment can influence later relationships and schemas even into adulthood [29].

Previous research has looked into the association between trauma symptoms and attachment patterns in adults. O’Connor and Elkilt (2008) [30] found that secure attachment was related to fewer PTSD symptoms. Other research has suggested that those with insecure attachment are more likely to have more trauma symptoms, particularly combat veterans [31]. A meta-analysis of the literature found that secure attachment was linked to lower levels of PTSD symptoms, and preoccupied and disorganized attachment patterns were correlated with a medium-sized effect on PTSD symptoms [32]. Less is known, however, about the same correlation in adolescents. Blum et al. (2022) [33] discovered that attachment insecurity was linked with overall childhood trauma symptoms in a group of young people. Additionally, gender might influence the relationships among attachment security, mental representations, and trauma symptoms.

### 1.3. Gender Differences in Trauma, Mental Representations, and Attachment Security

In recent years, gender has become more of a malleable concept, particularly among children and adolescents, with many young people identifying as nonbinary or gender-fluid [34]. While the consequences of gender fluidity and trauma have not been explored in depth, research has demonstrated binary gender disparities in relation to exposure to trauma and symptoms of PTSD [35,36]. It has been suggested that females are more prone to PTSD than males because of the different types of trauma they experience, with rape and sexual abuse being more prevalent in females [37]. Additionally, women are more likely to perceive the world as a dangerous place following a traumatic event than men, potentially due to heightened biological stress responses [38,39]. Moreover, young adolescent girls have been observed to be more inclined to internalize trauma, take the blame for it, and feel guilty when compared to boys [12]. These distinctions might be linked to differences in the way PTSD is expressed by gender.

Research suggests that males and females respond differently to trauma and demonstrate different symptoms related to PTSD. A study found that women might experience more feelings of guilt, while men might be more likely to express feelings of anger [40]. Additionally, military veterans have shown that men are more likely to become emotionally detached than women [41]. Regarding the 9/11 World Trade Center attack, men were more likely to have increases in cortisol due to re-experiencing symptoms, while women did not have the same reaction [42]. Despite these differences, secure attachment levels for both genders during early adolescence appear to be comparable [43].

Few studies have investigated any potential distinctions between males and females in terms of mental representations during adolescence. Data show that adolescent girls typically possess lower self-esteem and more unfavorable self mental representations than boys [44]. Additionally, girls with less ideal parental relationships and mental representations have been found to have weaker self-esteem and more negative self mental representations [45]. Attachment insecurity was associated with hyperarousal in young adolescent boys and re-experiencing and avoidance in young adolescent girls [33].

A single research study has been conducted to gauge the effects of attachment patterns, mental representations, and gender on the number of childhood trauma symptoms experienced by young adolescents. Ortigo et al. (2013) [46] looked into how object relations impact the link between attachment and PTSD symptoms in an adult group. Results showed that object relations mediate the relation between attachment and PTSD symptoms. Yet, the study did not explore the adolescent population or the differences in gender in detail.

### 1.4. Interventions for Traumatic Experiences and PTSD in Adolescents

While cognitive-behavioral therapy (CBT) and its variations are the most commonly used intervention approaches for PTSD [47], psychodynamic principles can also be used in interventions addressing PTSD, which provide interventionists with alternatives for individuals who do not respond to first-line interventions [48,49]. Psychodynamic therapy (PDT) has already been shown to be successful in treating symptoms of PTSD in adults (e.g., [50]). Two psychodynamically informed group interventions for adolescents include the STSA-A play-based group intervention, which was originally developed for preschool children, and MBT-G, which was originally developed for adults. The original STSA group intervention [51,52] consists of two phases: (1) students’ dictation of spontaneously generated stories (storytelling phase), and (2) students’ acting out these stories on a makeshift stage (story-acting phase). This group intervention has been shown to improve literacy skills, narrative comprehension, and social competence [53]. The STSA group intervention has been modified for an adolescent population [54]. 

STSA for Adolescents (STSA-A) allows for more control and input on the part of the student than the original STSA intervention [54]. In STSA-A, adolescents work together in small groups to convey and act out stories usually involving interpersonal conflict. Adolescents control who portrays each role and how the story is told. This psychodrama makes it easier to reflect and sublimate their experiences of close relationships so that they can be more easily understood. STSA-A differs from STSA because it allows adolescents to shape and reshape the narrative in a collaborative effort. Two group leaders facilitate the intervention, giving the group complete creative control over the story content as well as its portrayal. The repetitions of scenes to “get it just right” confirm the power of conveying unmetabolized feelings so that they can be “digested”—understood and ultimately verbalized. Because of its emphasis on creating narratives, STSA-A bears some resemblance to Narrative Exposure Therapy (NET), which in a meta-analysis has been shown to be effective at reducing trauma symptoms in adults [55].

Another group intervention that has been modified for work with young adolescents is MBT-G. MBT-G for adolescents [56,57] consists of sharing thoughts and feelings in the context of everyday social situations. In this setting, students improve their mentalization (the ability to understand ideas, intentions, and emotions of the self and others), which has been linked to more secure attachment, better affect regulation, and increased positive outcomes [58]. This approach is meant to be a playground of ideas and an exchange of experiences guided by a focus on activating, modeling, and experiencing the practice of mentalization in the context of the safety of a group environment with peers and two group leaders who attempt to co-regulate each other’s mentalizing and provide a model of relationships based on ambiguity and inquisitiveness [57]. The idea behind this group intervention is to create what in mentalization theory is described as “epistemic trust” [59], that is, a sense in the group that one’s thoughts and feelings matter to others. Epistemic trust is communicated and modeled by the group leaders both verbally and nonverbally, allowing for openness in the adolescents to new ways of relating. Helping individuals mentalize their traumatic experiences has been proposed as a strategy for restoring affect regulation in individuals with trauma symptoms [60].

### 1.5. Hypotheses

First, we hypothesized that adolescents who participated in STSA-A or MBT-G would demonstrate more positive affective valence in their mental representations of mother, father, self, and primary group leader from the first session in October (Time 1 [T1]) to the final session in May (Time 2 [T2]), regardless of intervention condition. Relatedly, we hypothesized that these adolescents would demonstrate increased attachment security. We also hypothesized that these adolescents would demonstrate lower severity of PTSD symptoms, as measured by total trauma, re-experiencing, avoidance, and hyperarousal. 

Second, we hypothesized that differences might exist in changes taking place between boys and girls, irrespective of intervention condition. We examined changes between T1 and T2 in boys and girls separately, combining intervention conditions. We remained agnostic about the existence and direction of gender differences in relation to changes in affective valence of mental representations, attachment security, and severity of trauma symptoms.

Third, we hypothesized that, when intervention conditions were examined separately, adolescents in both STSA-A and MBT-G would demonstrate more positive affective valence in their mental representations, increased attachment security, and lower severity of PTSD symptoms.

Fourth, we hypothesized that differences might exist in changes taking place between adolescents participating in STSA-A and adolescents participating in MBT-G. We remained agnostic about the existence and direction of differences in intervention condition in relation to changes in affective valence of mental representations, attachment security, and severity of trauma symptoms.

Fifth, we hypothesized that differences might exist in changes taking place between adolescents participating in STSA-A and adolescents participating in MBT-G for boys and girls separately. Again, we remained agnostic about the existence and direction of differences in intervention condition in relation to changes in affective valence of mental representations, attachment security, and severity of trauma symptoms for boys and girls separately.

## 2. Method

### 2.1. Participants

One-hundred nine students in eighth grade from a nonreligious private middle school, consisting mainly of ethnically and socioeconomically minoritized adolescents from New York City, participated in the study. The youth, ages 12 to 13, were primarily Latin American (35%), Asian (24%), African (18%), European (6%) and African American (11%) with the other 6% reporting either other or mixed ethnic backgrounds. Out of the participants, 44% (*n* = 48) identified as female, while none identified as nonbinary or transgender. We selected this age group because we believed that young adolescents would be cognitively mature enough to tolerate an hour-long group intervention, while at the same time not yet having been exposed to the challenges of high school.

Approximately 76% of the participants were first-generation Americans, with 82% coming from two-parent households. Sixty-three percent of the group leaders were female, while 58% were born in the US. The remaining group leaders originated from countries such as Australia, China, Switzerland, Serbia, and Italy. Over the course of four years (2015–2016, 2016–2017, 2017–2018, 2018–2019), 47.6% of a total of 229 eighth-grade adolescents attending this middle school took part in one of the two intervention conditions. Twenty-five students (45% of their cohort) participated in the first year, 24 students (39% of their cohort) in the second year, 42 students (68% of their cohort) in the third year, and 18 students (39% of their cohort) in the fourth year. Across all four years, 47.6% of the eighth-grade adolescents attending this middle school (*N* = 229) participated in one of the two intervention conditions.

### 2.2. Measures

#### 2.2.1. Child PTSD Symptom Scale (CPSS)

The CPSS [61] is a 24-question self-report questionnaire created to measure the frequency of PTSD symptoms in children and adolescents. It requires roughly 10 min to finish. The first part of the questionnaire consists of 17 questions, which are responded to on a 4-point Likert scale, with options ranging from 0 (“never”) to 3 (“five or more times a week”). This study comprised three sections, which focused on particular symptoms related to PTSD such as re-experiencing trauma (e.g., unpleasant thoughts), avoidance (e.g., emotional distance), and hyperarousal (e.g., difficulty concentrating). Total scores were calculated by summing the scores of each section (ranging from 0 to 51), with re-experiencing ranging from 0 to 15, avoidance from 0 to 21, and hyperarousal from 0 to 15. The measure was found to be valid, with a correlation of 0.80 when compared to the Child PTSD Reaction Index. In the present study, the CPPS demonstrated excellent internal consistency. T1 and T2 Cronbach’s alphas were calculated for each variable: re-experiencing (0.80, 0.83), avoidance (0.78, 0.90), hyperarousal (0.77, 0.86), and total (0.90, 0.94). Part 2 of the instrument was not included in the study.

#### 2.2.2. Object Relations Inventory (ORI) 

The ORI [62] measures the quality of an individual’s images of themselves and important people in their life. Participants are invited to describe certain individuals in an open-ended manner—in whatever way they choose. For this study, we gave four separate instructions to participants: “Describe your [mother, father, self, and primary group leader]”. We selected these four mental representations because of their importance to socioemotional well-being [19,21,63]. This procedure took approximately five minutes to complete, but participants were permitted as much time as they needed to write their responses. The ORI was designed to collect data on an individual’s knowledge and beliefs about themselves and other people, without providing benchmarks for comparison [19]. To determine the affective valence of these four written descriptions, a coding system was adapted specifically for this purpose.

To gauge the emotional valence of each individual’s four written ORI descriptions, we adapted the Positive and Negative Affect Schedule, Short Form (PANAS-SF; [64]). This assessment consists of 20 descriptions of how children have felt in the past week, with 10 being positive (e.g., proud) and 10 being negative (e.g., upset). In the PANAS-SF’s original form, respondents are asked to rate how they felt on a scale from 1 (“very slightly or not at all”) to 5 (“extremely”). The positive and negative descriptors are then added together to form a positive and negative affect score. The tool has been demonstrated to be dependable, with the coefficient alpha being 0.89 for positive affect and 0.85 for negative affect. After a one-week break, no noteworthy disparities were observed between scores, indicating its test–retest reliability. Moreover, its discriminant validity has been confirmed, as positive and negative affect scores account for only 1–5% of the variance of each other’s scores [64].

We used the PANAS-SF as a template for student coders to identify the positive and negative emotions expressed in the ORI’s written responses. For each written response, a positive (+1) or negative (−1) rating was assigned based on which descriptor from the PANAS-SF list most closely matched it. For instance, the written response “she is amazed at what I accomplished” would receive a +1 rating as it is similar to the positive descriptor “proud”, while the written response “she gets angry at me often” would be given a −1 rating as it is most similar to the negative descriptor “upset”. The affective valence of each written response was calculated by subtracting the negative affective valence score from the positive affective valence score for each ORI written response. Six psychology students, who were unaware of the study’s hypotheses, coded randomly assigned written responses to the prompts mother, father, self, and primary group leader, and interrater reliability was determined (intraclass correlation = 0.96).

### 2.3. Adolescent Attachment Questionnaire (AAQ)

The Adolescent Attachment Questionnaire (AAQ; [65]) was employed by the personnel of the school, including licensed therapists, social workers, and psychology interns who were in direct contact with the students, to assess the attachment quality of the participants. The staff filled out 37 items on a scale from 1 (not true) to 7 (very true) related to various characteristics of attachment patterns. The items that corresponded to each of the four attachment patterns (secure, preoccupied, dismissing, and incoherent/disorganized) were summed to create a continuous variable for each pattern, with higher scores indicating a greater degree of similarity to the attachment pattern. Only the attachment security variable (11 items) was used, with higher scores corresponding to higher levels of attachment security. In the present study, the AAQ attachment security variable demonstrated excellent internal consistency. T1 and T2 Cronbach’s alphas were calculated for attachment security (0.96, 0.96).

### 2.4. Procedure

The Institutional Review Board of the first author’s former university approved this study (15/05-310). The school principal notified students of the study and the accompanying intervention, which was advertised as having been integrated into the school curriculum for those interested. Students were informed that the school was offering support groups to discuss their feelings for any student who wanted to participate. These groups would continue for the duration of the school year. Students chose to take part in the study without any selection process based on prior traumatic experiences. Those who expressed interest in participating were given the chance to do so after obtaining a verbal assent from the student and written permission from the student’s legal guardian. Students and legal guardians consented to student participation in one of two group interventions for eight months as well as completion of a series of questionnaires in a group setting in October (T1) and May (T2). Students completed the ORI and CPSS in approximately 20 min in one sitting. The primary group leader completed the AAQ on each of her or his own participants to determine participants’ attachment security. The school principal also issued a letter authorizing student involvement in the research. Data on each child were obtained from school databases. Participants in the study were insured confidentiality. A study ID was given to each individual so that they could answer the questions without revealing their identities. Those who participated in the investigation did not receive any form of compensation.

**Figure 1:**
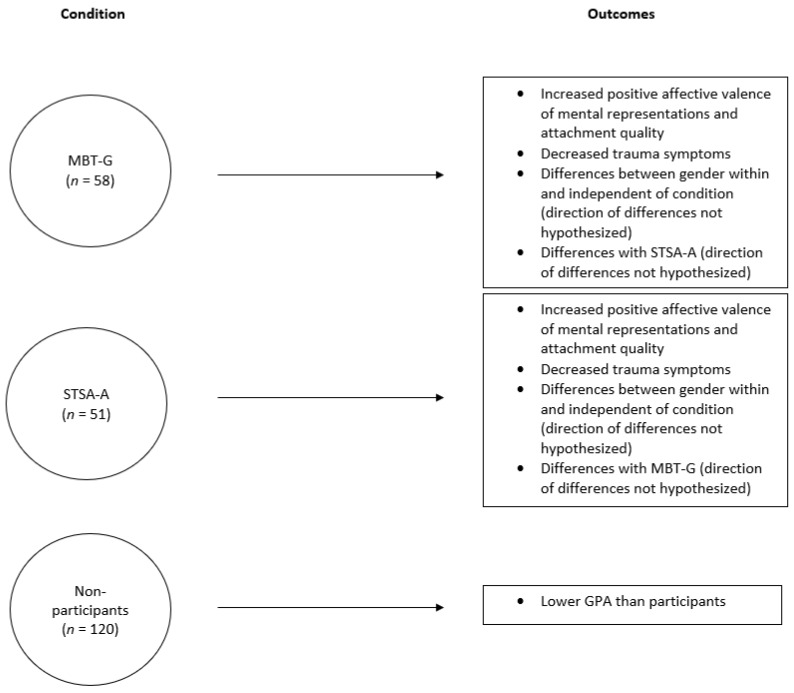
Study Model.

The school principal permitted student randomized assignment to two intervention conditions delivered in weekly, one-hour group interventions (STSA-A, MBT-G; previously described) regularly scheduled as part of the school curriculum (see Figure 1). Each October, the co-principal investigator explained to participants that the goal of the group intervention was to understand themselves and each other better to deal with stressors at home and at school. For STSA-A and MTB-G, there were two randomly assigned, gender-stratified groups. For each of the four years of this study, four groups were conducted—two STSA-A groups and two MBT-G groups. Each intervention condition included one all-boys group and one all-girls group; thus, two all-boys groups and two all-girls groups were conducted each year. Groups were gender-stratified to enhance the feelings of safety and permit participants to talk more freely about anything, including sexual issues [66]. Each group consisted of approximately eight members. A total of 21 students dropped out of the group interventions within the first two weeks of the academic year. These 21 students were identified as nonparticipants, along with the students who never enrolled in the group interventions (*n* = 120).

To test whether participants differed from nonparticipants, we collected semester grade-point averages (GPAs) from all students in January and in June at the end of the academic year. An independent-samples *t*-test indicated that in January, participants’ GPA was significantly lower than nonparticipants’ GPA, *t*(227) = −2.17, *p* = 0.031. Thus, it seems plausible to suggest that the academically weaker students were more attracted to participate in these groups than other students. By June, however, participants’ GPA was no longer significantly lower than nonparticipants’ GPA, *t*(227) = −1.81, *ns*, which suggests that participation in a group intervention helped to boost GPA. No significant differences in GPA were found between intervention conditions in January or June; thus, we will not report any further on GPA.

A senior-level clinician supervised the group leaders, who were licensed clinical psychologists, social workers, and clinical psychology externs. Each group consisted of a primary and an assistant group leader. To insure adherence to the manuals of the two group interventions, the supervisor conducted weekly 1.5 h supervision groups for the four group leaders in each intervention condition. The eight group leaders available to lead the four groups each year (two STSA-A groups, two MBT-G groups) were randomly assigned to one of the two intervention conditions to prevent allegiance effects. The two developers of STSA-A and MBT-G certified the supervisor in the conduct of both STSA-A and MBT-G and made themselves frequently available to consult with the supervisor about any issues that arose in the groups.

Students remained in the group for the full academic year. The ORI, the AAQ, and the CPSS were administered at T1 (prior to the first group session) and T2 (after the final group session) to assess students’ progress and to test the hypotheses.

### 2.5. Data Analysis

To test hypotheses 1 through 3, we used a series of paired-samples *t*-tests, pairing the nine T1 and T2 outcome variables. To test hypotheses 4 and 5, we used a series of independent-samples *t*-tests. We first created nine change outcome variables by subtracting each T1 outcome variable from each T2 variable. The two intervention conditions—STSA-A and MBT-G—served as the dichotomous independent variable. All missing data were replaced using the expectation-maximization (EM) technique in SPSS^®^ Version 28.0.1.0. Cohen’s *d* was used to calculate effect size. The conventional guidelines presented by Ref. [67] were used in interpreting these effect sizes; a Cohen’s *d* of 0.20 indicates a small effect, 0.50 indicates a medium effect, and 0.80 indicates a large effect.

## 3. Results

### 3.1. Changes in Affective Valence of Mental Representations, Attachment Security, and Childhood Trauma Symptoms from T1 to T2

From T1 to T2, participants reported changes in the affective valence of mental representations (see Table 1). From T1 to T2, the affective valence of paternal mental representations decreased significantly (*t* [108] = 2.48, *p* = 0.015, *d* = 0.48), but attachment security increased significantly (*t* [108] = −5.05, *p* < 0.001, *d* = 0.97). Regarding childhood trauma symptoms from T1 to T2 (see Table 1), participants experienced decreased levels of total childhood trauma symptoms (*t* [108] = 4.94, *p* < 0.001, *d* = 0.95). There were also significant decreases from T1 to T2 for re-experiencing trauma (*t* [108] = 3.22, *p* = 0.002, *d* = 0.61), avoidance (*t* [108] = 4.20, *p* < 0.001), and hyperarousal (*t* [108] = -4.11, *p* < 0.001, *d* = 0.79).

### 3.2. Gender Changes in Affective Valence of Mental Representations, Attachment Security, and Childhood Trauma Symptoms from T1 to T2

Boys reported two changes in the affective valence of mental representations from T1 to T2 (see Table 2). From T1 to T2, boys reported decreases in the affective valence of maternal (*t* [60] = 2.33, *p* = 0.023, *d* = 0.60) and paternal (*t* [60] = 2.54, *p* = 0.014, *d* = 0.65) mental representations. Boys also experienced increases in attachment security (*t* [60] = −4.25, *p* < 0.001, *d* = 1.10). Girls did not report any changes in affective valence of mental representations from T1 to T2 (see Table 2), but they did experience increases in attachment security (*t* [60] = −2.77, *p* = 0.008, *d* = 0.71). 

Regarding childhood trauma symptoms, boys and girls reported similar decreases from T1 to T2. For boys (see Table 2), decreases of total childhood trauma symptoms were found from T1 to T2 (*t* [60] = 3.95, *p* < 0.001, *d* = 1.02). Boys also experienced decreases from T1 to T2 in re-experiencing trauma (*t* [60] = 2.51, *p* = 0.015, *d* = 0.65), avoidance (*t* [60] = 3.30, *p* = 0.002, *d* = 0.83), and hyperarousal (*t* [60] = 4.03, *p* < 0.001, *d* = 1.04). In addition, from T1 to T2, girls (see Table 2) experienced decreases in total childhood trauma symptoms (*t* [47] = 2.95, *p* = 0.005, *d* = 0.86), re-experiencing trauma (*t* [47] = 2.03, *p* = 0.048, *d* = 0.59), and avoidance (*t* [47] = 2.60, *p* = 0.013, *d* = 0.75) but not hyperarousal.

### 3.3. Intervention Condition Changes in Affective Valence of Mental Representations, Attachment Security, and Childhood Trauma Symptoms from T1 to T2

Differences existed regarding affective valence of mental representations based on therapy condition (see Table 3). For the MBT-G condition, affective valence of primary group leader mental representations decreased from T1 to T2 (*t* [57] = 2.84, *p* = 0.006, *d* = 0.76), and attachment security increased from T1 to T2 (*t* [57] = −3.16, *p* = 0.003, *d* = 0.82). For the STSA-A condition, affective valence of paternal mental representations decreased from T1 to T2 (*t* [50] = 2.47, *p* = 0.017, *d* = 0.70), and attachment security increased from T1 to T2 (*t* [50] = −4.18, *p* < 0.001, *d* = 1.18).

Both STSA-A and MBT-G intervention conditions experienced decreases in childhood trauma symptoms from T1 to T2 (see Table 3). In the MBT-G condition, participants exhibited decreases from T1 to T2 in total childhood trauma symptoms (*t* [57] = 3.23, *p* = 0.002, *d* = 0.86) as well as re-experiencing trauma (*t* [57] = 2.31, *p* = 0.03, *d* = 0.59), avoidance (*t* [57] = 2.63, *p* = 0.01, *d* = 0.71), and hyperarousal (*t* [57] = 2.11, *p* = 0.04, *d* = 0.56). Similar results were found for participants in the STSA-A condition; from T1 to T2, they also reported lower total childhood trauma symptoms (*t* [50] = 3.73, *p* < 0.001, *d* = 1.05), re-experiencing trauma (*t* [50] = 2.28, *p* = 0.03, *d* = 0.63), avoidance (*t* [50] = 3.28, *p* = 0.002, *d* = 0.92), and hyperarousal (*t* [50] = 3.79, *p* < 0.001, *d* = 1.07).

### 3.4. Differences between Conditions in Changes of Affective Valence of Mental Representations, Attachment Security, and Childhood Trauma Symptoms from T1 to T2

Intervention condition had an impact on the magnitude of change of affective valence of primary group leader mental representations (see Table 4). Participants in the STSA-A condition had a significantly greater change in affective valence of primary group leader mental representations than participants in the MBT-G condition did from T1 to T2 (*t* [107] = −3.05, *p* = 0.003, *d* = 0.59). In other words, the affective valence of primary group leader mental representations of the STSA-A participants became more positive over time, while the affective valence of primary group leader mental representations of the MBT-G participants actually became more negative over time.

### 3.5. Boys’ and Girls’ Differences between Conditions in Changes of Affective Valence of Mental Representations, Attachment Security, and Childhood Trauma Symptoms from T1 to T2

These results were consistent for both boys (see Table 5) and girls (see Table 6). Boys in the STSA-A condition had a marginally significantly more positive change in affective valence of primary group leader mental representations than boys in the MBT-G condition from T1 to T2 (*t* [59] = −1.95, *p* = 0.056, *d* = 0.51) such that the affective valence of primary group leader mental representations of the STSA-A boys became more positive over time, while the affective valence of primary group leader mental representations of the MBT-G boys became more negative over time. Girls in the STSA-A condition also had a significantly greater change in affective valence of primary group leader mental representations than girls in the MBT-G condition from T1 to T2 (*t* [46] = −2.33, *p* = 0.025, *d* = 0.68) such that the affective valence of primary group leader mental representations of the STSA-A girls became more positive over time, while the affective valence of primary group leader mental representations of the MBT-G girls became more negative over time.

## 4. Discussion

This study demonstrated that two different group interventions implemented in an urban middle-school setting were successful in improving students’ attachment security and reducing trauma symptoms in typically developing young adolescents from ethnically and socioeconomically minoritized backgrounds. Students benefit from school-based group intervention [68,69], but few studies have addressed the effectiveness of psychodynamically oriented school-based group interventions. The two interventions offer two different strategies for resolving trauma symptoms. In STSA-A, participants created narratives that often enacted stressful life events that seemed to serve as containment for their traumatic experiences [54,70]. In MBT-G, participants explored their own and others’ mental states in a supportive, trusting environment [57]. We are proposing that while equally efficacious, one intervention might be more efficacious for certain groups of adolescents than the other (see later discussion).

The fact the students participating in both the STSA-A and MBT-G intervention conditions were not previously identified as having socioemotional difficulties and yet demonstrated positive changes suggests that school administrators can make these two group interventions widely available as primary prevention measures. Even low-risk students need opportunities for emotional expression, affect regulation, and reflection on interpersonal experiences, which could prevent children from becoming at risk or even clinically disordered. These psychodynamically oriented group interventions are not designed to replace other popular group interventions but rather to add to the list of successful interventions that might be attempted, especially if students show less enthusiasm for other group interventions [71]. Attachment security [72] and trauma reduction [73] are both associated with favorable outcomes, which could protect adolescents from needing psychiatric services in adulthood.

Interestingly, examining the total sample, as the affective valence of paternal mental representations became significantly more negative, attachment security significantly improved. We view these two changes as interrelated. According to Main (1995) [74], the dismissing attachment pattern (a subcategory of attachment insecurity) often includes idealized versions of childhood attachment figures, which protect the individual from chronic unconscious feelings of rejection by these attachment figures. Attachment security represents the ability to view attachment figures more realistically while deriving comfort and soothing from their internalized presence. It is plausible that as the study participants (particularly boys and particularly STSA-A participants) were beginning to permit narratives of paternal rejection to come to the surface and integrate these narratives with more positive narratives of paternal comfort, primary group leaders (who rated the AAQ) observed simultaneous improvements in participants’ attachment security. The paternal mental representations were thus “de-idealized” and replaced with more realistic and comforting mental representations.

While girls in both intervention conditions did not show any changes in affective valence of mental representations over time, boys exhibited decreases in affective valence of maternal and paternal mental representations over time, instead of an expected increase. This finding might be related to an increased desire for individuation among boys in early adolescence. Males are more likely than females to seek independence from their parents during this phase of development [75]. At the beginning of adolescence, a shift begins to occur as their relational and societal commitments begin to gain ascendancy over their commitments to their parents [76]. Successful intervention with adolescents sometimes involves disengaging from parents and developing new mental representations [77]. By reducing some involvement with parents, especially for students with more significant clinical impairments, students can have more involvement with others outside their family dynamics such as friends and romantic partners. Allowing more freedom to explore other relational dynamics stimulates changes in parental schemata, particularly the capacity to incorporate more negative mental representations into an integrated schema [78]. The incorporation of more negative mental representations might have decreased the affective valence of parental representations for boys more than girls during the intervention because of the increased desire for individualization among boys during this time period.

Unexpected changes over time for the affective valence of mental representations were also identified. When considering the two intervention conditions, participants in the STSA-A condition experienced decreases in affective valance of paternal mental representations over time, while participants in the MBT-G condition experienced decreases in affective valence of primary group leader mental representations over time. The changes in mental representations of their parents and themselves, and perhaps also their primary group leaders (if they have made an emotional connection), can affect how they respond to traumatic experiences after the intervention. Attachment security increased in both intervention conditions—no small feat, considering the unique and complex challenges these children of immigrants needed to work through to begin to view their parents and school authority figures as sources of support and comfort.

As suggested earlier, perhaps paternal mental representations and primary group leader mental representations became de-idealized in the STSA-A and MBT-G conditions, respectively. Intervention for young adolescents sometimes involves incorporation of negative parental attributes into schemata [78]. The objective of the STSA-A protocol was to create a story and attempt to understand different characters’ perspectives, regardless of whether the characters are affectively positive or negative. It is possible that during STSA-A specifically, participants became better able to understand the intentions of paternal figures, which could have been negative. In Latin American immigrant families especially, fathers can typically represent figures of authoritarianism in these highly patriarchal societies [79,80]. Fathers can play a large role in adolescent mental health, considering that more paternal involvement in parenting can result in better mental health outcomes [81].

Herzog (2013) [82] proposed the idea of “father hunger,” in which fathers are necessary to help male children and adolescents synthesize emotions and desires such as aggression. Growing up, if a boy is “starved” of an emotional or physical paternal presence, he can struggle to synthesize these complicated emotions later in life, sometimes developing psychopathology. Feeling chronically overwhelmed can hinder fathers from mentalizing their sons’ emotional needs, which could in turn produce this father hunger and arrest the development of mentalization and the capacity for co-regulation and overall affect regulation in situations of stress. In addition, father hunger can also lead to resentment toward the absent father and more negatively affectively valenced paternal mental representations. Telling and acting out stories in which the father is featured might bring more negative paternal feelings to the surface, making them more consciously accessible and thus more available for integration with more positive paternal feelings by the end of the STSA-A group intervention.

Differences in changes of affective valence of mental representations from T1 to T2 were also different than hypothesized for the primary group leader mental representations, as the positive change in the STSA-A condition was significantly greater than the negative change in the MBT-G condition. Similar differences between changes in affective valence of primary group leader mental representations were also reported for both boys and girls. No significant differences in changes from T1 to T2 were reported for childhood trauma symptoms based on condition or gender, supporting the hypothesis.

The decrease in affective valence of primary group leader mental representations in the MBT-G condition might be accounted for by MBT-G group leaders’ experience level. Research has indicated that successful intervention using MBT-G requires a high level of skill and proficiency from a clinician to establish epistemic trust with students [83]. Given that some of the group leaders were novice clinical externs, they might not have had the necessary experience to establish epistemic trust with these young adolescents. In addition, although we did not examine differences in enjoyment, previous research suggests that adolescents enjoy group interventions more focused on the arts than traditional group therapy [84,85]. Thus, MBT-G group leaders, who assumed a more traditional role, might have given their participants less freedom to make the group their own, and the affective valence of primary group leader mental representations the MBT-G participants formed of them consequently diminished.

## 5. Limitations

Several limitations of this study need to be mentioned. The sample size was small and lacked socioeconomic diversity. Although group leaders attended weekly 1.5 h supervision meetings, and the supervisor had immediate and routine access to the creators of both group interventions if technical questions arose, we did not systematically check adherence to the manuals of the two group interventions. Most school settings do not permit video or audio-recording equipment in school settings for research or clinical purposes.

We also used a relatively crude coding system that consisted of positive and negative affects of the four mental representations. Although the PANAS-SF is established to accurately detect positive and negative affective valence, its use in the open-ended ORI meant that certain responses did not have a corresponding descriptor from the PANAS-SF. For instance, the phrase “my mom is a nurse” does not have a descriptor from the PANAS-SF. Hence, these types of responses were not coded for affective valence. Despite this drawback, we are confident that we were able to identify the affective valence of most responses using this approach. Further research should assess the precision of the responses and create clearer categories for positive and negative responses. Coding responses for level of differentiation-relatedness (e.g., checking for incoherence and confusion in the self representation; [86]) and mentalizing (e.g., reflective functioning; [87]) would be two possible avenues to pursue.

## 6. Recommendations for Future Research

Future research should examine the efficacy of these two group interventions on older adolescents (e.g., ages 14–19). In addition, future studies should examine the processes of change in the attachment security and the affective valence of mental representations in MBT-G and STSA-A. Although both intervention conditions were effective at increasing attachment security and reducing trauma symptoms, STSA-A participants demonstrated more positive changes in affective valence of primary group leader mental representations compared to MBT-G participants, which might indicate that STSA-A would uniquely benefit adolescents who have had challenges with authority figures. STSA participants “own” the dramatic process, which perhaps creates a perception that their group leaders are validating of their creativity and need for control. By contrast, MBT-G group leaders restricted their participants’ mode of group expression to an exclusively verbal dimension, which might feel occasionally invalidating. STSA-A participants’ developing more positively affectively valenced primary group leader mental representations could provide some added value in functioning, especially if these improvements generalize to significant others such as parents and teachers. Previous research has indicated that more positive therapist mental representations are associated with greater frequency of therapy and a stronger working alliance [88].

MBT-G, on the other hand, might be more effective for adolescents who have negative affectively valenced paternal mental representations, protecting paternal mental representations from too much de-idealization. This conclusion is supported by the result that STSA-A showed a significant decrease in affective valence of paternal representations. How this process works is not currently known and should be further understood to enhance outcomes. Furthermore, future research could examine whether an integrated approach utilizing both STSA-A and MBT-G would be effective in changing the affective valence of both paternal and primary group leader mental representations. Perhaps young adolescents wary of authority figures could benefit from STSA-A for eight months until they are ready for MBT-G to promote their mentalization skills. Such intervention scaffolding might boost representational change beyond what either intervention could accomplish alone.

## 7. Conclusions

In conclusion, this study demonstrated the efficacy of both STSA-A and MBT-G, as participants in both intervention conditions demonstrated significant increases in attachment security and decreases in trauma symptoms over eight months for young adolescents in a school setting. Considering over half of children experience a potentially traumatic event [3,89], these group interventions can serve as a form of primary prevention in mitigating future effects of trauma symptoms. Notably, a review of the literature indicates that successful psychological interventions made in early adolescence are maintained throughout life [90]. Due to its collaborative, hands-on approach, STSA-A might be more effective at maintaining a working alliance with group leaders. On the other hand, due to its awakening of participants’ curiosity about others’ mental states and motivations, MBT-G might be more effective at protecting relationships with fathers. The findings strongly suggest, however, that school psychologists in school settings can implement both STSA-A and MBT-G for effective improvements in attachment security and reduction of trauma symptoms in typically developing young adolescents.

## Figures and Tables

**Table 1:** Changes in affective valence of mental representations, attachment security, and childhood trauma symptoms from T1 to T2.

	T1		T2		Significance
	*M*	*SD*	*M*	*SD*	
ORI Variables					
Mother	1.95	2.90	1.54	1.60	
Father	1.51	2.50	0.95	2.01	T1 > T2 **
Self	1.57	2.69	1.29	2.04	
Group Leader	1.73	1.08	1.58	1.25	
Secure Attachment	3.90	1.42	4.34	1.45	T2 > T1 ***
CPSS Variables					
Total Trauma	28.12	9.57	24.16	10.54	T1 > T2 ***
Re-Experiencing	8.33	3.15	7.28	3.42	T1 > T2 **
Avoidance	11.06	4.33	9.41	4.86	T1 > T2 ***
Hyperarousal	8.72	3.30	7.47	3.30	T1 > T2 ***

*N* = 109. ** *p* < 0.01. *** *p* < 0.001.

**Table 2:** Gender changes in affective valence of mental representations, attachment security, and childhood trauma symptoms from T1 to T2.

	Boys ^a^				Significance		Girls ^b^			Significance
	T1		T2		Boys	T1		T2		Girls
	*M*	*SD*	*M*	*SD*		*M*	*SD*	*M*	*SD*	
ORI Variables										
Mother	2.61	3.21	1.79	1.44	T1 > T2 *	1.11	2.21	1.23	1.74	
Father	2.21	2.65	1.37	1.92	T1 > T2 *	0.61	1.98	0.40	2.00	
Self	2.11	3.00	1.58	1.87		0.88	2.08	0.91	2.20	
Group Leader	1.71	1.07	1.65	1.20		1.76	1.11	1.49	1.33	
Secure Attachment	3.66	1.51	4.21	1.59	T2 > T1 ***	4.20	1.25	4.52	1.25	T2 > T1 **
CPSS Variables										
Total Trauma	26.92	8.96	22.54	9.74	T1 > T2 ***	29.64	10.19	26.23	11.25	T1 > T2 **
Re-Experiencing	8.00	2.90	6.96	3.35	T1 > T2 *	8.76	3.43	7.69	3.51	T1 > T2 *
Avoidance	10.55	3.69	8.87	4.31	T1 > T2 **	11.71	5.00	10.09	5.44	T1 > T2 *
Hyperarousal	8.37	3.33	6.70	2.90	T1 > T2 ***	9.17	3.19	8.54	3.54	

^a^ *n* = 61. ^b^ *n* = 48. * *p* < 0.05. ** *p* < 0.01. *** *p* < 0.001.

**Table 3:** Intervention condition changes in affective valence of mental representations, attachment security, and childhood trauma symptoms from T1 to T2.

	MBT-G ^a^				Significance		STSA-A ^b^			Significance
	T1		T2		MBT-G	T1		T2		STSA-A
	*M*	*SD*	*M*	*SD*		*M*	*SD*	*M*	*SD*	
ORI Variables										
Mother	1.79	2.95	1.37	1.53		2.16	2.87	1.73	1.66	
Father	1.11	2.21	0.85	1.96		1.96	2.75	1.06	2.07	T1 > T2 *
Self	1.27	2.84	1.12	1.19		1.91	2.51	1.41	2.24	
Group Leader	1.83	1.06	1.32	1.23	T1 > T2 **	1.62	1.12	1.88	1.23	
Secure Attachment	3.78	1.47	4.20	1.44	T2 > T1 **	4.03	1.36	4.50	1.46	T2 > T1 ***
CPSS Variables										
Total Trauma	27.75	10.32	24.38	11.46	T1 > T2 *	28.52	8.74	23.92	9.49	T1 > T2 ***
Re-Experiencing	8.41	3.42	7.36	3.83	T1 > T2 *	8.25	2.85	7.31	2.94	T1 > T2 *
Avoidance	10.83	4.63	9.50	5.25	T1 > T2 *	11.31	4.00	9.30	4.41	T1 > T2 **
Hyperarousal	8.51	3.50	7.62	3.61	T1 > T2 **	8.97	3.01	7.31	2.93	T1 > T2 ***

^a^ *n* = 58. ^b^ *n* = 51. * *p* < 0.05. ** *p* < 0.01. *** *p* < 0.001.

**Table 4:** Differences between conditions in changes of affective valence of mental representations, attachment security, and childhood trauma symptoms from T1 to T2.

	MBT-G ^a^		STSA-A ^b^	
	*M*	*SD*	*M*	*SD*
ORI Variables				
Mother	−0.40	2.80	−0.42	2.55
Father	−0.26	2.08	−0.90	2.59
Self	−0.09	2.94	−0.50	2.93
Group Leader *	−0.51	1.37	0.26	1.27
Secure Attachment	0.42	1.02	0.47	0.81
CPSS Variables				
Total Trauma	−3.38	7.95	−4.61	8.83
Re-Experiencing	−1.15	3.80	−0.94	2.94
Avoidance	−1.33	3.85	−2.01	4.38
Hyperarousal	−0.89	3.21	−1.66	3.13

^a^ *n* = 58. ^b^ *n* = 51. * *p* < 0.01.

**Table 5:** Boys’ differences between conditions in changes of affective valence of mental representations, attachment security, and childhood trauma symptoms from T1 to T2.

	MBT-G ^a^		STSA-A ^b^	
	*M*	*SD*	*M*	*SD*
ORI Variables				
Mother	−0.93	2.97	−0.71	2.54
Father	−0.56	1.93	−1.14	3.15
Self	−0.41	3.28	−0.66	3.21
Group Leader ^†^	−0.36	1.01	0.27	1.49
Secure Attachment	0.47	1.09	0.63	0.92
CPSS Variables				
Total Trauma	−3.53	9.31	−5.32	7.93
Re-Experiencing	−1.02	3.66	−1.05	2.75
Avoidance	−1.20	4.20	−2.20	3.68
Hyperarousal	−1.31	3.47	−2.06	2.98

^a^ *n* = 32. ^b^ *n* = 29. ^†^ *p* = 0.061.

**Table 6:** Girls’ differences between conditions in changes of affective valence of mental representations, attachment security, and childhood trauma symptoms from T1 to T2.

	MBT-G ^a^		STSA-A ^b^	
	*M*	*SD*	*M*	*SD*
ORI Variables				
Mother	0.25	2.48	−0.04	2.58
Father	0.11	2.24	−0.57	1.61
Self	0.31	2.47	−0.29	2.58
Group Leader *	−0.70	1.72	0.25	0.93
Secure Attachment	0.36	0.95	0.27	0.60
CPSS Variables				
Total Trauma	−3.18	6.06	−3.67	10.01
Re-Experiencing	−1.31	4.04	−0.79	3.46
Avoidance	−1.50	3.46	−1.76	5.23
Hyperarousal	−0.38	2.86	−1.13	3.31

^a^ *n* = 26. ^b^ *n* = 22. * *p* < 0.05.

## Data Availability

Due to privacy and ethical considerations, data are unavailable for re-analysis.
